# Effect of Composite Viscosity, Cavity Type, and Placement Technique on Microleakage in Stamp Technique Restorations: An In Vitro Study

**DOI:** 10.3390/biomimetics11060382

**Published:** 2026-06-01

**Authors:** Ayşenur Yazım, Cemile Kedici Alp, Ceyda Sarı

**Affiliations:** 1 Department of Restorative Dentistry, Faculty of Dentistry, Gazi University, Ankara 06490, Türkiye; cemilealp@gazi.edu.tr; 2 Department of Restorative Dentistry, Faculty of Dentistry, Istanbul Medipol University, Istanbul 34083, Türkiye; ceyda.gundogdu@medipol.edu.tr

**Keywords:** stamp technique, biomimetic dentistry, bulk-fill composites, microleakage

## Abstract

The biomimetic restoration of occlusal morphology aims to replicate the natural form and function of dental tissues while preserving structural integrity. The stamp technique enables accurate reproduction of occlusal anatomy in posterior restorations; however, the extent to which different composite systems maintain marginal integrity and reduce microleakage when this technique is applied remains unclear. This in vitro study evaluated the microleakage of bulk-fill resin composites with different viscosities applied using the stamp technique. A total of 120 extracted human molars (*n* = 10 per group) were prepared with standardized Class I and Class II cavities and restored using SonicFill™, VisCalor, or Filtek™ Bulk-Fill composites, applied either in a single or two increments. After thermocycling, specimens were dye-penetrated, sectioned, and analyzed using ImageJ. Data were analyzed by two-way ANOVA and Tukey’s HSD test (*p* < 0.05). In Class I cavities, occlusal microleakage differed significantly among materials (*p* = 0.001), with SonicFill™ showing the lowest values. In Class II cavities, gingival microleakage was significantly affected by both material and placement technique (*p* = 0.001). Incremental placement significantly reduced gingival microleakage for SonicFill™ and Filtek™ Bulk-Fill. Material viscosity and placement strategy influence marginal adaptation under stamp technique conditions. Low-viscosity bulk-fill composites demonstrated improved sealing, while incremental placement enhanced marginal integrity, particularly in Class II cavities.

## 1. Introduction

Biomimetic dentistry aims to restore dental structures using materials that mimic the aesthetic, functional, and mechanical properties of natural tissues such as enamel, dentin, and cementum, while also reestablishing the tooth’s natural biomechanics [1]. These restorative approaches emphasize the preservation of healthy dental tissues and utilize advanced materials to achieve durable, visually satisfactory outcomes [2]. Composites and adhesive systems are employed to restore carious lesions in a minimally invasive manner, maintaining the tooth’s structural integrity [3].

Among biomimetic restoration techniques, the stamp technique is a highly precise method, particularly for posterior restorations, that replicates the original occlusal anatomy [4]. In this technique, the morphology of the occlusal surface is recorded prior to cavity preparation, thereby ensuring the precise recreation of the natural occlusal form [5]. It is particularly advantageous in cases where the occlusal anatomy and marginal ridges remain intact despite carious involvement. Compared with conventional layering methods, which are time-consuming and require clinical expertise, occlusal morphology can be standardized, and treatment time can be reduced using the stamp technique [6].

Posterior restorations aim for clinical ease, aesthetics, and long-term success. The marginal integrity of dental composites is influenced by numerous factors, including polymerization shrinkage, polymerization techniques, differences in thermal expansion coefficients, operator skill and application technique, material composition, occlusal stress, finishing and polishing quality, and material aging. These factors can potentially cause voids and lead to problems such as microleakage, sensitivity, and a shortened restoration lifespan. Bulk-fill composites reduce polymerization shrinkage stress, interlayer gaps, and procedure time, while improved material properties help minimize microleakage and extend restoration longevity [7]. Although incremental placement techniques may reduce polymerization shrinkage stress, they are generally considered technique-sensitive and may increase the risk of void formation or contamination between layers [8]. Advances in composites have increased the use of bulk-fill, prompting research into alternative techniques. Recent bulk-fill composites have introduced modified monomer systems to control polymerization stress. Proper material flow and adaptation to cavity walls are important factors in minimizing void formation and improving restoration integrity in bulk-fill restorations [8]. Filtek™ One Bulk Fill incorporates high-molecular-weight aromatic urethane dimethacrylate (AUDMA) and a patented addition–fragmentation monomer (AFM) designed to undergo stress-relieving fragmentation reactions during network formation, thereby reducing polymerization shrinkage stress without decreasing mechanical performance [9]. One approach to improving adaptation is reducing viscosity through vibration. SonicFill™, a sonically activated bulk-fill composite, can be placed in a single 5 mm increment. Sonic energy reduces viscosity, improving cavity adaptation, and it regains optimal viscosity for shaping when activation stops [10]. Recent studies have also investigated the advantages of preheating composites and have demonstrated improvements in viscosity, surface hardness, and workability. Preheating has been associated with improved flow properties without compromising mechanical strength and has shown potential to reduce microleakage in restorations [11].

Despite advances in restorative materials, microleakage remains an unresolved issue that negatively affects the long-term success of restorations [12]. Therefore, it remains a critical parameter in evaluating the clinical efficacy of restorative techniques and materials [13]. The stamp technique facilitates the creation of tooth anatomy and can shorten finishing time; however, its application, particularly in cavities with a high C factor, may increase the risk of polymerization shrinkage when a single composite layer is applied. This effect can also be critical in Class II cavities where the restoration margin is located on dentin or cementum, which may increase susceptibility to microleakage.

However, most existing studies are limited to case reports and do not adequately evaluate clinically important parameters, such as marginal fit and microleakage. These mainly focus on its use in Class I restorations, with only a few studies on Class II cavities. In previous SEM-based evaluations of the stamp technique, restorations were mainly performed using incremental placement protocols [14]. In contrast, the present study additionally evaluated bulk-fill placement strategies using composites with different viscosity behaviors, allowing assessment of how material flow and adaptation may be influenced under stamp application conditions.

In restorations performed with the stamp technique, the placement of composite under occlusal pressure may compromise marginal integrity, increasing microleakage. There are few studies on the effect of the stamp technique on microleakage in composites with different viscosities, particularly when applied to both Class I and Class II cavities. Unlike conventional microleakage studies that evaluate bulk-fill composites alone, the present study specifically investigated how occlusal pressure generated during the stamp technique may interact with composite viscosity and polymerization. It was hypothesized that low-viscosity materials may better dissipate the pressure applied by the stamp and improve adaptation to cavity walls before gelation, whereas high-viscosity composites may exhibit restricted flow under the same conditions, potentially increasing interfacial stress concentration and microleakage, particularly in high C-factor cavities. Under standardized cavity configurations, evaluation of different viscosity-related material behaviors may provide additional insight into the influence of placement and cavity adaptation during stamp technique application. In this study, the following three materials were selected to represent three different viscosity strategies: sonic activation (SonicFill), thermo-viscous preheating (VisCalor), and traditional high-viscosity bulk-fill composite (Filtek One). These materials were selected to represent three clinically relevant viscosity-modification approaches currently used in restorative dentistry rather than the entire spectrum of bulk-fill composites. SonicFill employs sonic energy to temporarily reduce viscosity during placement, and VisCalor utilizes thermal preheating to enhance flowability, whereas Filtek One represents a conventional high-viscosity bulk-fill composite formulation. This study aimed to evaluate the effect of composite viscosity and placement technique on microleakage when the stamp technique is applied in standardized Class I and Class II cavities.

This study has three null hypotheses. The first null hypothesis was that there was no significant difference in occlusal microleakage among SonicFill™, VisCalor, and Filtek™ One bulk-fill composites used to restore Class I cavities with the stamp technique. The second null hypothesis was that there was no significant difference in gingival microleakage between different bulk-fill composite materials applied using the stamp technique in Class II cavities. The third null hypothesis was that there was no significant difference in microleakage between bulk and layered application techniques of the same composite material in both Class I and Class II cavity restorations.

## 2. Materials and Methods

This in vitro study on the microleakage of bulk-fill composites applied with the stamp technique was approved by the Ethics Committee of Gazi University Faculty of Dentistry (06.04.2023-E.630221) and conducted in accordance with the Declaration of Helsinki.

### 2.1. Tooth Selection and Preparation of the Occlusal Index (Stamp Technique)

The sample size of the study was determined using a power analysis performed with G*Power software (version 3.1.9.6). For the 12-group study design, a significance level of 0.05, a statistical power of 90%, and an effect size of 0.50 were assumed. The selected effect size (0.50) was estimated according to previous in vitro microleakage studies with comparable methodologies evaluating restorative materials and marginal adaptation [15,16] and Cohen’s recommendations for medium effect sizes in biological research. This value was considered appropriate for detecting clinically meaningful differences among restorative materials under standardized experimental conditions. Considering the potential for specimen loss, 10 specimens were included in each group. Accordingly, 120 caries-free, intact maxillary and mandibular molars were selected.

**Figure 1:**
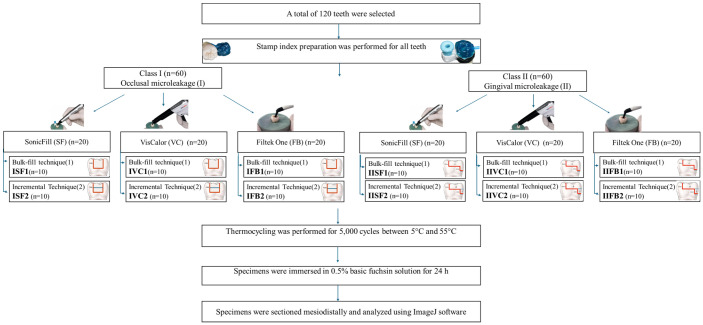
Flowchart of the experimental procedure.

**Figure 2:**
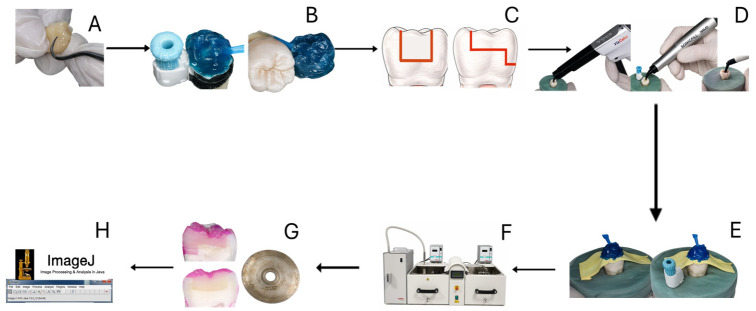
Representative photographs illustrating the experimental procedure. (**A**) Initial preparation of extracted teeth. (**B**) Fabrication of the occlusal index using a gingival barrier material. (**C**) Standardized Class I and Class II cavity designs. (**D**) Placement of VisCalor, Filtek One, and SonicFill composite resins. (**E**) Stamp repositioning procedures in Class I and Class II restorations. (**F**) Thermocycling procedure. (**G**) Mesiodistal sectioning of specimens. (**H**) Microleakage evaluation using ImageJ software.

The stages of the experimental procedure, including the study flowchart and group allocation, are illustrated in Figure 1. The procedural steps are illustrated in Figure 2.

Prior to cavity preparation, the Opaldam gingival barrier (Ultradent Products, South Jordan, UT, USA) was passively placed by a single operator. The gingival barrier was applied in a smooth, even layer, extending approximately 1 mm beyond the occlusal surface to cover the occlusal anatomy. The gingival barrier was secured with a bonding brush and light-cured for 20 s using a D-Light Pro (GC Europe, Leuven, Belgium) with a light intensity of 1200 mW/cm^2^. Restorative procedures were completed immediately after the Stamp index was prepared for each tooth.

For Class II cavities, occlusal indices were created after placement of a Palodent circumferential matrix band (Dentsply Sirona, Charlotte, NC, USA) to ensure proper proximal contour and accurate stamp adaptation. During restoration, the matrix band was stabilized using the matrix retainer system throughout composite placement and stamp application.

### 2.2. Class I Cavity Preparation Design

In the first six groups, standardized Class I cavities were prepared with a width corresponding to one-third of the bucco-lingual distance, measured using a digital caliper (Insize 1112-150, INSIZE Inc., Loganville, GA, USA). Cavities were centered along the bucco-lingual axis, with the central fossa located at the midpoint of the cavity width. A cavity depth of 4 mm was measured from the occlusal sulcus using a periodontal probe, and 2 mm of intact tooth structure was preserved mesially and distally.

### 2.3. Class II Cavity Preparation Design

**Figure 3:**
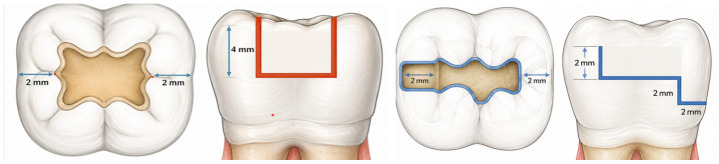
Illustrative representation of the Class I and Class II cavity preparation procedure.

In the remaining six groups, standardized Class II mesio-occlusal cavities were prepared with the same bucco-lingual width, preserving 2 mm of distal tooth structure, depth 2 mm beyond the deepest occlusal sulcus, and a proximal box positioned 2 mm below the pulpal floor, with a standardized step width of 2 mm measured using a periodontal probe. The cavity designs are schematized in Figure 3.

### 2.4. Group Assignment and Restorative Materials

Class I and II cavities were prepared in 60 teeth, with 4 maxillary and 6 mandibular molars per group. Tooth dimensions were measured using a digital caliper (Insize 1112-150, Insize Inc., USA) and analyzed using one-way ANOVA to confirm that there were no significant size differences among groups (*p* > 0.05). Teeth were then randomly allocated into groups of 10. Materials and their properties are summarized in Table 1.

### 2.5. Restorative Technique

**Figure 4:**
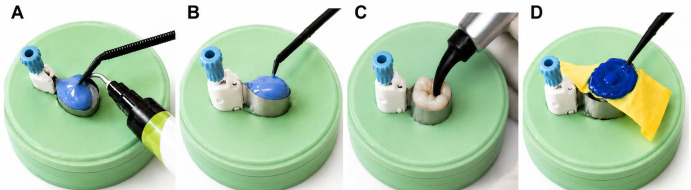
Representative images of occlusal stamp application. (**A**) Application of the gingival barrier after placement of the matrix band. (**B**) Fabrication of the occlusal stamp. (**C**) Composite resin application. (**D**) Formation of the occlusal morphology using Teflon tape.

All restorations were performed by a single operator. Cavities were prepared using fissure burs (G&Z Instrumente GmbH, Lustenau, Austria) at 40,000 rpm (Combident unit, KaVo, Biberach, Germany) under water cooling. The burs were replaced after every five cavity preparations. All cavities were etched with 37% phosphoric acid for 30 s on enamel and 15 s on dentin, rinsed thoroughly, and gently air-dried. Single Bond Universal (3M ESPE, St. Paul, MN, USA) was actively applied to enamel and dentin surfaces for 20 s using a scrubbing motion, gently air-dried for 5 s to ensure solvent evaporation, and light-cured for 10 s using a D-Light Pro LED curing unit(GC Europe N.V., Leuven, Belgium) After composite placement, a Teflon tape was positioned over the uncured composite to prevent adhesion between the restorative material and the stamp. In Class II cavities, restorations were completed after the matrix band was placed. The stamp impression was then passively repositioned, as described in previous studies [4,17]. It was aligned according to the original occlusal anatomy and lightly pressed with a finger to recreate the occlusal morphology. To stabilize the surface form, a short light-curing process was performed through the Teflon tape, after which excess composite material was carefully removed using hand instruments. All restorations were light-cured using a D-Light Pro curing unit at 1200 mW/cm^2^. The composite was polymerized according to the manufacturer’s instructions, with the light tip positioned vertically and as close as possible to the restoration surface. Finishing and polishing of all restorations were performed with cooling underwater using fine-grit diamond burs and polishing disks (Sof-Lex, 3M, St. Paul, MN, USA). The occlusal stamp application procedure is illustrated in Figure 4.

Group ISF1: SonicFill Bulk-fill composite restorations were applied using the SonicFill handpiece (Kerr Corporation, Orange, CA, USA) designed by the manufacturer. The composite cartridge was attached to the SonicFill handpiece, and the material was placed into the cavity under sonic activation. The pre-prepared occlusal index was placed onto the composite surface with controlled pressure, and polymerization was completed. In the first six groups, except for differences in application technique, the remaining restorative steps were identical to those described for Group ISF1.

Group ISF2: SonicFill™ was performed incrementally in two 2 mm layers. After the first layer of composite was applied, it was condensed with a hand instrument under light pressure.

Group IVC1: VisCalor was performed in a single increment. VisCalor Bulk-fill restorations were applied after preheating, as recommended by the manufacturer. Composite cartridges were heated to a target temperature of 68 °C using the VisCalor Dispenser device (VOCO GmbH, Cuxhaven, Germany). After heating, the cartridge was removed from the device and placed into the cavity within 15–20 s. The pre-prepared occlusal index was placed onto the composite surface with controlled pressure, and polymerization was completed.

Group IVC2: VisCalor was performed incrementally in two layers. After the first layer of composite was applied, it was condensed with a hand instrument under light pressure.

Group IFB1: Filtek™ One Bulk-fill was performed in a single 4 mm increment; restoration was completed as in Group ISF1.

Group IFB2: Filtek™ One Bulk-fill was performed incrementally in two layers of 2 mm, each polymerized. After the first layer of composite was applied, it was condensed with a hand instrument under light pressure.

Group IISF1: SonicFill™ was performed in a single increment to Class II cavities. After placing the matrix band, the composite was covered with Teflon tape. The pre-prepared occlusal index was placed onto the composite surface with controlled pressure, and polymerization was completed. In the second set of six groups, except for differences in application technique, the remaining restorative steps were identical to those described for Group IISF1.

Group IISF2: SonicFill™ was performed incrementally in Class II cavities in two 2 mm layers. After the first layer of composite was applied, it was condensed with a hand instrument under light pressure.

Group IIVC1: VisCalor was performed in a single increment to Class II cavities; restoration was completed as in Group IISF1.

Group IIVC2: VisCalor was performed incrementally in Class II cavities. After the first layer of composite was applied, it was condensed with a hand instrument under light pressure.

Group IIFB1: Filtek™ One Bulk-fill was performed in a single 4 mm increment to Class II cavities.

Group IIFB2: Filtek™ One Bulk-fill was performed incrementally in two 2 mm layers in Class II cavities. After the first layer of composite was applied, it was condensed with a hand instrument under light pressure.

### 2.6. Microleakage Evaluation

**Figure 5:**
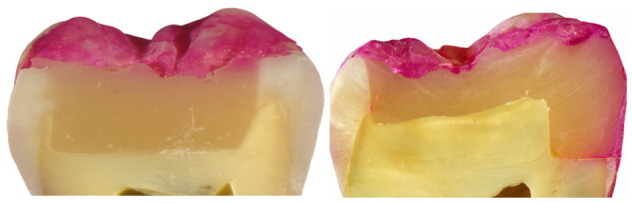
Representative images demonstrating microleakage patterns in Class I and Class II restorations.

After cavity preparation, specimens were incubated at 37 °C for 24 h, thermocycled 5000 times between 5 °C and 55 °C (20 s dwell), and immersed in 0.5% basic fuchsin for 24 h. After immersion in basic fuchsin, the teeth were rinsed under running water. Crowns were separated from roots, sectioned mesio-distally under water cooling (Presi, Mecatome, T 201 A, Eybens, France). Images were obtained using a digital camera (Canon EOS 70D DSLR Canon Inc., Tokyo, Japan)) with a macro lens under standardized conditions. The images were imported into ImageJ software (version 1.54; National Institutes of Health, Bethesda, MD, USA) and calibrated prior to analysis using a millimeter scale included in each image. The linear extent of dye penetration along the tooth–restoration interface was measured and divided by the total cavity wall length of the corresponding restoration to obtain the microleakage ratio for each specimen. For each tooth, the highest microleakage value from the two sections was selected for statistical analysis. Representative microleakage patterns observed in Class I and Class II restorations are presented in Figure 5.

### 2.7. Statistical Analysis

The obtained data were analyzed using IBM SPSS Statistics 22 software. The normality of the parameters was assessed using the Kolmogorov–Smirnov and Shapiro–Wilk tests, and all parameters were found to be normally distributed. Two-way ANOVA was used to evaluate the combined effects of material and application method on occlusal and gingival microleakage levels. Post hoc multiple comparisons were performed using the Tukey HSD test. Effect size was evaluated using partial eta squared (η^2^p), and 95% confidence intervals were calculated for the relevant parameters. Statistical significance was accepted at *p* < 0.05.

## 3. Results

The effects of material type and application method on occlusal microleakage in Class I restorations were evaluated using a two-way ANOVA analysis. The corresponding statistical results are presented in Table 2.

A statistically significant difference in occlusal microleakage was observed among the evaluated materials (F = 115.856; *p* = 0.001; η^2^p = 0.811), indicating a large effect size. No significant difference was found between application methods (F = 3.547; *p* = 0.065; η^2^p = 0.062). In addition, the interaction effect between material type and application method was not statistically significant (F = 0.380; *p* = 0.686; η^2^p = 0.014), indicating a negligible interaction effect size.

Occlusal microleakage differed significantly among materials (*p* = 0.001). No significant differences were observed between methods (*p* = 0.065) or for the material–method interaction (*p* = 0.686). Detailed results are shown in Table 3.

In bulk application, occlusal microleakage differed significantly among materials (*p* = 0.001). Group IFB1 showed higher microleakage than Groups ISF1 and IVC1 (*p* = 0.001), and IVC1 was higher than ISF1 (*p* = 0.009). In the layered application, differences were also significant (*p* = 0.001). Group IFB2 had higher microleakage than Groups ISF2 and IVC2 (*p* = 0.001), and IVC2 was higher than ISF2 (*p* = 0.001). No difference was found between bulk and layered applications for SonicFill (*p* = 0.546) or VisCalor (*p* = 0.492). Filtek One bulk (IFB1) showed higher microleakage than the layered application (IFB2) (*p* = 0.021).

The effects of material type and application method on gingival microleakage were evaluated using a two-way ANOVA analysis. The corresponding results are presented in Table 4.

A statistically significant difference in gingival microleakage was observed among the evaluated materials (F = 27.299; *p* = 0.001; η^2^p = 0.503), indicating a large effect size. Application methods also showed a statistically significant effect on gingival microleakage levels (F = 20.798; *p* = 0.001; η^2^p = 0.278), indicating a moderate-to-large effect size. In addition, the interaction between material type and application method was statistically significant (F = 10.662; *p* = 0.001; η^2^p = 0.283), indicating a moderate-to-large interaction effect.

Gingival microleakage was significantly affected by material (*p* = 0.001), method (*p* = 0.001), and their interaction (*p* = 0.001). Detailed results are in Table 5.

In Class II cavities with bulk application, gingival microleakage differed significantly between materials (*p* = 0.001). Group IIFB1 showed higher microleakage than Groups IISF1 and IIVC1 (*p* = 0.001), with no difference between IISF1 and IIVC1 (*p* = 0.626). In layered applications, microleakage also differed (*p* = 0.001). Group IISF2 had lower microleakage than Groups IIFB2 and IIVC2 (*p* = 0.001; *p* = 0.006), with no difference between IIVC2 and IIFB2 (*p* = 0.289). For SonicFill, bulk (IISF1) showed higher leakage than layered application (IISF2) (*p* = 0.014). VisCalor showed no difference between bulk and layered application (*p* = 0.166). Filtek One had higher microleakage in bulk (IIFB1) than in layered application (IIFB2) (*p* = 0.003).

## 4. Discussion

The present study evaluated the effect of composite viscosity and placement technique on microleakage in Class I and Class II restorations performed using the stamp technique. The findings demonstrated that both material type and application technique influenced marginal adaptation, particularly at the gingival margins of Class II cavities. These differences may be associated with the viscosity-related flow behavior and polymerization characteristics of the evaluated materials. Filtek One Bulk Fill contains AFMs, which are associated with reduced polymerization shrinkage and improved marginal adaptation [18]. In contrast, VisCalor and SonicFill employ different viscosity-modifying strategies: thermoviscous preheating and sonic activation, respectively [19,20,21]. These materials were specifically selected to represent three clinically relevant viscosity-related approaches currently used in bulk-fill restorative systems: conventional high-viscosity bulk-fill behavior (Filtek One Bulk Fill), thermo-viscous preheating (VisCalor), and sonic-activated viscosity reduction (SonicFill).

The stamp technique, a recent alternative for posterior restorations, offers advantages over traditional layering by preserving occlusal morphology, particularly in teeth with intact occlusal contours [3]. Zhu et al. reported that this technique maintains its accuracy in shallow cavities (1–2 mm), but its effectiveness in deeper cavities remains uncertain [17]. Bud et al. found no significant differences in microleakage between the stamp and the traditional techniques [22]. Rathi et al. reported that the stamp technique allowed accurate reproduction of anatomical occlusal contours with a significantly shorter operative time than conventional restorative techniques, without negatively affecting microleakage, marginal adaptation, or void formation [23]. The stamp technique applied with Teflon tape is the more commonly used method in the clinic. In a study comparing the accuracy of occlusal anatomy, results were obtained showing that the stamp technique applied with Teflon tape was superior [24], and therefore, the stamp technique was applied using Teflon tape. Previous studies have shown that the etch-and-rinse approach is associated with reduced microleakage rates, whereas the self-etch mode of universal adhesives may lead to increased void formation in both bulk and layered restorations. Therefore, in the present study, the universal adhesive was applied using the etch-and-rinse mode [25,26].

To reduce polymerization shrinkage stress and, consequently, minimize microleakage, incremental placement of composite resins has long been recommended. This technique may allow more favorable stress distribution and improved adaptation of the restorative material to cavity walls, potentially contributing to restoration longevity. However, increased time requirements and the risk of contamination between layers have led to the development of simplified restorative approaches in clinical practice. Bulk-fill composites are widely used because they require fewer procedural steps and allow faster application procedures [27]. Although bulk-fill materials may exhibit increased polymerization shrinkage stress, previous studies have reported comparable clinical performance, bond strength, and marginal adaptation to those of conventional composites, even in high C-factor cavities [28]. The findings of the present study support the proposed mechanistic hypothesis that viscosity-related flow behavior under stamp-induced pressure may influence marginal adaptation. SonicFill and VisCalor, which undergo temporary viscosity reduction through sonic activation or preheating, demonstrated lower microleakage values than the conventional high-viscosity bulk-fill composite. Previous studies have shown that viscosity reduction may improve cavity adaptation and reduce polymerization stress by enhancing composite flow before gelation [19,20,29]. Sonic activation has been reported to improve material adaptation through transient viscosity reduction and increased flowability [16,20], while preheating has been associated with improved flow characteristics and stress distribution during polymerization [19,30]. Under the compressive effect generated during the stamp technique, materials with improved flow behavior may better adapt to cavity walls and dissipate polymerization stresses before reaching the gel phase, thereby contributing to reduced marginal microleakage.

Thermal cycling is an important aging procedure in microleakage studies because it simulates temperature changes that occur under oral conditions. Gale et al. suggested that approximately 10,000 thermal cycles correspond to one clinical year [31]. Therefore, in the present study, 5000 cycles were performed to simulate approximately six months of aging. Specimens were then immersed in basic fuchsin solution, which is considered a suitable and effective dye penetration method [32]. Microleakage analysis was subsequently performed using ImageJ software to provide an objective evaluation of dye penetration [33]. Several studies have discussed the advantages and limitations of dye penetration and advanced three-dimensional imaging methods in microleakage evaluation [13,32,34]. Ernst et al. also demonstrated that dye penetration analysis provides reliable information on marginal integrity and reported a good correlation between dye penetration findings and SEM observations in restorative margins [32]. Similarly, Zanatta et al. reported that micro-CT analysis yielded significantly lower microleakage values than conventional stereomicroscopy and concluded that micro-CT may underestimate dye penetration due to insufficient radiographic contrast between dental tissues, restorative materials, and tracer solutions [34]. Although micro-CT enables non-destructive three-dimensional analysis, dye penetration methods remain sensitive and clinically relevant for evaluating marginal leakage pathways. Therefore, in the present study, dye penetration combined with digital ImageJ analysis was preferred because the primary aim was to compare the relative microleakage performance of different bulk-fill composites and placement techniques under standardized experimental conditions rather than to obtain a complete volumetric characterization of the restoration–tooth interface.

In the present study, IFB1 and IFB2 demonstrated higher microleakage values than the other restorative materials evaluated in Class I cavities. These findings are consistent with the in vivo study conducted by Morsy et al., who reported that polymerization shrinkage stress and the risk of microleakage may increase in high C-factor Class I restorations. In their study, Filtek One demonstrated less favorable long-term performance, whereas SonicFill and VisCalor exhibited improved marginal adaptation and lower microleakage values, which were associated with their lower viscosity characteristics [35]. In bulk-fill composites, filler loading plays an important role in determining viscosity, polymerization shrinkage behavior, mechanical properties, and cavity adaptation. Filtek One Bulk Fill contains a comparatively lower filler loading (approximately 76.5 wt%/58.5 vol%) than other composites and instead relies on advanced monomer chemistry, including AUDMA and AFMs, to reduce polymerization stress while maintaining favorable mechanical properties [36]. SonicFill and VisCalor Bulk Fill exhibit relatively high filler loading values of approximately 81% and 83%, respectively, which may contribute to reduced polymerization shrinkage and improved mechanical performance. However, increased filler loading may also increase material viscosity and potentially compromise adaptation to cavity walls during placement. To compensate for this limitation, SonicFill utilizes sonic activation to temporarily reduce viscosity during placement, whereas VisCalor Bulk Fill employs a thermoviscous preheating approach to enhance flowability, polymerization, and shrinkage kinetics and fracture toughness of bulk-fill resin composites. An in vitro study comparing VisCalor and SonicFill in Class I restorations reported that VisCalor demonstrated superior marginal adaptation; while this was attributed to increased flowability following preheating, it was considered that SonicFill required greater operator experience [37]. These findings differ from those of the present study, which showed that ISF1 and ISF2 exhibited lower microleakage values than IVC1 and IVC2. This inconsistency may be related to differences in material handling and placement behavior, as SonicFill provides continuous sonic activation during placement, which may temporarily reduce viscosity and improve cavity adaptation. In contrast, the flow behavior of thermoviscous composites such as VisCalor may be affected by temperature loss after removal from the heating device, potentially compromising marginal adaptation during placement. Previous studies have reported that the temperature of preheated composites decreases rapidly after removal from the heating unit, thereby requiring prompt clinical application [38]. Consequently, delays during placement or between increments may negatively influence adaptation quality and may partially explain the higher microleakage observed in layered VisCalor applications, consistent with the findings of Faverato et al. [39]. Therefore, the first null hypothesis was rejected.

In Class I cavities restored using the stamp technique, single-layer groups exhibited higher microleakage than double-layer groups. Although a layered application does not completely eliminate microleakage, it reduces it more effectively. No significant differences in occlusal microleakage between bulk and layered applications of ISF1vs ISF2 and IVC1 vs. IVC2 were observed in Class I cavities. However, IFB1 resulted in significantly greater microleakage than IFB2, possibly because its high viscosity may benefit from condensation forces during incremental placement. Our findings suggest that low-viscosity composites, such as SonicFill or VisCalor, may be more suitable for bulk applications in Class I cavities using the stamp technique.

Although Class II restorations also include an occlusal component, the present study specifically focused on gingival microleakage because the gingival margin is the most technique-sensitive region regarding adhesion and polymerization stress development, particularly when located on dentin or cementum [21,40,41]. Therefore, gingival leakage was considered the primary outcome parameter for evaluating marginal adaptation in Class II restorations.

In Class II cavities restored using bulk-fill techniques, significant differences in gingival microleakage were observed among the tested materials. IIFB1 exhibited higher gingival microleakage compared to IISF1 and IIVC1. This difference may be related to the viscosity-reducing mechanisms of SonicFill and VisCalor, which facilitate access to and adaptation at the gingival margins. Previous studies have reported that Filtek One may exhibit reduced marginal adaptation after aging. Similarly, Mosharrafian et al. reported lower microleakage with SonicFill in primary teeth, attributing this to sonic activation, which improved material flow and cavity adaptation [42]. Andrade et al. found that VisCalor demonstrated better marginal adaptation than Filtek One in Class II restorations, and that this was related to the viscosity reduction achieved through thermal activation [43]. However, the effects of preheating on marginal adaptation remain controversial in the literature. Guarneri et al. reported that insufficient temperature at the cartridge tip during application could negatively affect marginal adaptation [44], while Alves de Sá et al. found that preheating did not significantly improve gingival adaptation [45]. In this study, IIVC1 reduced microleakage compared to IIFB1. Goda et al. and Yang et al. reported reduced microleakage in heated composites compared to those used at room temperature, supporting the findings of this study [18,46]. The findings suggest that gingival adaptation in Class II cavities may be more sensitive to both material viscosity behavior and placement technique than occlusal adaptation. Therefore, the second null hypothesis was rejected.

In Class II cavities restored with a layered application, significant differences in gingival microleakage were seen. IISF2 showed less leakage than IIVC2 and IIFB2; no difference was found between IIVC2 and IIFB2. For high-viscosity materials like Filtek One, initial condensation with a plugger may improve adaptation and reduce microleakage. Akalın et al. reported SonicFill as durable and clinically safe due to its deep polymerization capacity and low shrinkage stress [47], while Goracci et al. demonstrated homogeneous polymerization even in deeper increments, supporting its bulk-fill use [48]. However, complete elimination of microleakage appears unlikely regardless of the restorative material or technique used. Dalia et al. emphasized that microleakage is influenced not only by material properties but also by the structural characteristics of the bonded dental substrate [40]. In particular, the thinner and less mineralized enamel in the cervical region may increase susceptibility to leakage, which may explain the greater difficulty in achieving optimal bonding at the gingival margin of Class II cavities compared with occlusal areas. These findings are supported by in vitro studies demonstrating increased microleakage in cervical regions [41]. Therefore, meticulous adhesive application and careful material adaptation remain critical, especially at the gingival margin.

SonicFill maintains marginal adaptation after thermomechanical loading due to its mechanical strength and low shrinkage stress but requires caution in deep cavities to ensure adequate polymerization [33]. Campos et al. reported that SonicFill had the highest marginal adaptation pre- and post-thermomechanical loading in Class II cavities, attributed to its fluid consistency and low shrinkage stress [49]. IISF1 gingival microleakage was significantly higher than IISF2. Alsafii et al. reported that SonicFill performed optimally at a thickness of 2–4 mm; however, the risk increased when the application exceeded 4 mm [50]. In this study, SonicFill demonstrated the lowest microleakage in both bulk and layered applications of Class II stamp restorations; however, layered applications, which were statistically significant, further improved the outcomes.

Torles et al. showed that VisCalor provides effective polymerization and hardness in deep restorations [51]. Though bulk applications showed more leakage, no significant difference in gingival leakage was found between IIVC1 and IIVC2, possibly because layered polymerization reduces shrinkage and leakage.

This study corroborated that layering improves performance with the stamp technique for Filtek One. Filtek One Bulk-fill in Class II restorations exhibited significantly more gingival microleakage IIFB1 vs. IIFB2. Kim et al. reported better marginal adaptation with layered Filtek Bulk-fill and noted that vibration during layering improved adaptation and reduced voids [52]. Terada et al. noted that Filtek One Bulk-fill may not fully polymerize in thick layers, which reduces its hardness and stability [53]. Hassanain et al. observed that microhardness decreases with depth in bulk-fill composites and advocated layered application for clinical reliability [54].

From a biomimetic perspective, the success of restorative procedures depends not only on reproducing occlusal morphology but also on mimicking the biomechanical behavior of natural tooth structures. While the stamp technique enables accurate reproduction of occlusal anatomy, factors such as marginal adaptation and microleakage also play an important role in evaluating restoration success. Materials demonstrating improved adaptation may contribute to more favorable interfacial stress distribution at the tooth–restoration interface, potentially resulting in biomechanical behavior. In addition, incremental placement may allow a more controlled polymerization process, thereby improving interfacial integrity.

Several limitations of the present study should be considered. Although dye penetration analysis is a widely used, clinically relevant method for evaluating microleakage, it provides only two-dimensional information from selected sections of the restoration. Therefore, the total extent and distribution of leakage pathways may not be fully represented. Three-dimensional imaging techniques, such as micro-CT, could provide a more comprehensive evaluation of internal voids, interfacial adaptation, and leakage propagation throughout the restoration interface. Consequently, the findings of the present study should be interpreted within the limitations of two-dimensional leakage assessment. Due to the absence of adjacent teeth in Class II cavities, wedges were not applied, which may have affected gingival adaptation. The restorations were evaluated under completely dry laboratory conditions, and the effect of oral moisture on adhesive performance was not investigated. Furthermore, no comparison was made with conventional non-stamp restorative approaches. The pressure applied during stamp adaptation could not be quantitatively standardized and may have influenced composite adaptation and marginal integrity. One limitation of this study is that the rheological properties of the tested materials were not directly evaluated. Therefore, future studies including direct rheological analysis are needed to better understand the relationship between material viscosity and microleakage. Another limitation of the present study is that the occlusal pressure applied during stamp positioning was not quantitatively standardized. Although previous studies have described the use of digital pressure during stamp application, the magnitude of the applied force has not been clearly defined in the current literature. Future clinical and long-term studies would better establish the clinical validity of these findings. These findings may contribute to improved material selection and restorative strategies in clinical practice. Furthermore, the results may support future studies aimed at developing advanced bulk-fill restorative materials with improved marginal adaptation and long-term clinical performance.

## 5. Conclusions

Successful restoration requires preservation of the tooth–restoration interface integrity and adequate marginal adaptation. Within the limitations of this in vitro study, SonicFill demonstrated lower microleakage values in both Class I and Class II cavities restored using the stamp technique. The thermoviscous composite VisCalor did not demonstrate lower microleakage values than the conventional bulk-fill composite when applied in bulk in Class II cavities. In contrast, Filtek One Bulk-Fill demonstrated higher microleakage values when applied in bulk to both of the restored Class I and Class II cavities. Overall, the findings suggest that material viscosity and placement technique may influence microleakage behavior in restorations performed using the stamp technique. Further clinical studies are required to confirm the clinical relevance of these observations.

## Figures and Tables

**Table 1:** The materials and chemical compositions.

Restorative Material	Content	Type	Manufacturer
**SonicFill 2 (SF)**	Filler (% wt.): 83.5, SiO_2_, glass, oxide Matrix: Bis-GMA, TEGDMA, Bis-EMA	Sonic activated Bulk-fill	Kerr (Orange, CA, USA)
**VisCalor (VC)**	Filler (% wt.): 83 Matrix: Bis-GMA, aliphatic dimethacrylate	Thermo-viscous Bulk-fill	VOCO GmbH, (Cuxhaven, Germany)
**Filtek™ One Bulk-fill (FB)**	Filler (% wt.): 76.5, YbF_3_, zirconium, silica Matrix: AUDMA, AFM, UDMA, DDDMA	Conventional Bulk-fill	3M™ ESPE (St. Paul, MN, USA)
**Scotchbond™ Universal**	MDP phosphate monomer, Dimethacrylate resins, HEMA, Acid copolymer, Ethanol, Water, Initiator, Silan, Silica Filler, EDMAB	Universal Adhesive	3M™ ESPE (St. Paul, MN, USA)
**I-DENTAL I-Gel**	-	%37 Phosphoric Acid	I-Dental (Siauliai, Lithuania)
**Opaldam**	Methacrylate-based resin	Gingival barrier	Ultradent Products Inc. (South Jordan, UT, USA)

Abbreviations: Bis-GMA, Bisphenol A-glycidyl methacrylate; TEGDMA, Triethylene glycol dimethacrylate; Bis-EMA, Ethoxylated bisphenol A dimethacrylate; UDMA, Urethane dimethacrylate; YbF_3_, Ytterbium trifluoride; HEMA, 2-Hydroxyethyl methacrylate; MDP, 10-Methacryloyloxydecyl dihydrogen phosphate; EDMAB, Ethyl 4-dimethylaminobenzoate.

**Table 2:** Two-way ANOVA analysis of the effects of material and application method on occlusal microleakage.

Occlusal Microleakage	Type III Sum of Squares	df	Mean Square	F	*p*	Partial Eta Squared
Material	0.192	2	0.096	115.856	0.001 *	0.811
Method	0.003	1	0.003	3.547	0.065	0.062
Material × Method	0.001	2	0	0.38	0.686	0.014

Two-way ANOVA test. * *p* < 0.05.

**Table 3:** Evaluation of the combined effect of material and application method on the level of occlusal microleakage.

Occlusal Microleakage Ratio (I)	Bulk Technique (1)	Incremental Technique (2)	*p*
	Mean ± SD (95% CI)	Mean ± SD (95% CI)	
**SonicFill (SF)**	0.079 ± 0.03 ^Aa^ (0.054–0.104)	0.071 ± 0.02 ^Aa^ (0.055–0.087)	0.546
**VisCalor (VC)**	0.128 ± 0.04 ^Ba^ (0.098–0.158)	0.117 ± 0.03 ^Ba^ (0.098–0.136)	0.492
**Filtek One (FB)**	0.223 ± 0.02 ^Ca^ (0.206–0.240)	0.200 ± 0.02 ^Cb^ (0.188–0.212)	0.021 *
***p***	0.001 *	0.001 *	

Two-way ANOVA test. * *p* < 0.05. Distinct capital letters signify variations among materials, whereas distinct lowercase letters denote variations among applications.

**Table 4:** An evaluation of the combined effect of material and application method on the level of gingival microleakage.

Gingival Microleakage	Type III Sum of Squares	df	Mean Square	F	*p*	Partial Eta Squared
Material	0.523	2	0.261	27.299	0.001 *	0.503
Method	0.199	1	0.199	20.798	0.001 *	0.278
Material × Method	0.204	2	0.102	10.662	0.001 *	0.283

Two-way ANOVA test. * *p* < 0.05.

**Table 5:** Evaluation of the combined effect of material and application method on the gingival microleakage level.

Gingival Microleakage Ratio (II)	Bulk Technique (1)	Incremental Technique (2)	*p*
	Mean ± SD (95% CI)	Mean ± SD (95% CI)	
**SonicFill (SF)**	0.194 ± 0.04 ^Aa^ (0.165–0.223)	0.153 ± 0.02 ^Ab^ (0.136–0.170)	0.014 *
**VisCalor (VC)**	0.248 ± 0.03 ^Aa^ (0.224–0.273)	0.223 ± 0.04 ^Ba^ (0.192–0.254)	0.166
**Filtek One (FB)**	0.535 ± 0.22 ^Ba^ (0.378–0.692)	0.255 ± 0.06 ^Bb^ (0.209–0.301)	0.003 *
***p***	0.001 *	0.001 *	

Two-way ANOVA test. * *p* < 0.05. Distinct capital letters signify variations among materials, whereas distinct lowercase letters denote variations among applications.

## Data Availability

The datasets generated and/or analyzed during the current study are available from the corresponding author upon reasonable request.

## Abbreviations

The following abbreviations are used in this manuscript:

AFM	Addition–fragmentation monomer
ANOVA	Analysis of variance
Bis-EMA	Ethoxylated bisphenol A dimethacrylate
Bis-GMA	Bisphenol A-glycidyl methacrylate
C-factor	Configuration factor
EDMAB	Ethyl 4-dimethylaminobenzoate
HEMA	2-Hydroxyethyl methacrylate
HSD	Honestly Significant Difference
IBM SPSS	International Business Machines Statistical Package for the Social Sciences
MDP	10-Methacryloyloxydecyl dihydrogen phosphate
NIH	National Institutes of Health
SD	Standard deviation
SiO_2_	Silisyum dioksit
SPSS	Statistical Package for the Social Sciences
TEGDMA	Triethylene glycol dimethacrylate
UDMA	Urethane dimethacrylate
YbF_3_	Ytterbium trifluoride
